# Threat Response System: Parallel Brain Processes in Pain vis-à-vis Fear and Anxiety

**DOI:** 10.3389/fpsyt.2018.00029

**Published:** 2018-02-20

**Authors:** Igor Elman, David Borsook

**Affiliations:** ^1^Boonshoft School of Medicine, Wright State University, Dayton VA Medical Center, Dayton, OH, United States; ^2^Harvard Medical School, Center for Pain and the Brain, Boston Children’s Hospital, Massachusetts General Hospital, McLean Hospital, Boston, MA, United States

**Keywords:** amygdala, cognitive, fear, nociception, sensory, unconscious

## Abstract

Pain is essential for avoidance of tissue damage and for promotion of healing. Notwithstanding the survival value, pain brings about emotional suffering reflected in fear and anxiety, which in turn augment pain thus giving rise to a self-sustaining feedforward loop. Given such reciprocal relationships, the present article uses neuroscientific conceptualizations of fear and anxiety as a theoretical framework for hitherto insufficiently understood pathophysiological mechanisms underlying chronic pain. To that end, searches of PubMed-indexed journals were performed using the following Medical Subject Headings’ terms: pain and nociception plus amygdala, anxiety, cognitive, fear, sensory, and unconscious. Recursive sets of scientific and clinical evidence extracted from this literature review were summarized within the following key areas: (1) parallelism between acute pain and fear and between chronic pain and anxiety; (2) all are related to the evasion of sensory-perceived threats and are subserved by subcortical circuits mediating automatic threat-induced physiologic responses and defensive actions in conjunction with higher order corticolimbic networks (e.g., thalamocortical, thalamo-striato-cortical and amygdalo-cortical) generating conscious representations and valuation-based adaptive behaviors; (3) some instances of chronic pain and anxiety conditions are driven by the failure to diminish or block respective nociceptive information or unconscious treats from reaching conscious awareness; and (4) the neural correlates of pain-related conscious states and cognitions may become autonomous (i.e., dissociated) from the subcortical activity/function leading to the eventual chronicity. Identifying relative contributions of the diverse neuroanatomical sources, thus, offers prospects for the development of novel preventive, diagnostic, and therapeutic strategies in chronic pain patients.

## Introduction

Acute pain is essential for survival by avoidance of tissue damage and by promotion of healing. This is not so for chronic (i.e., lasting more than 3 months) pain (1) that has no beneficial value (2). By afflicting over 100 million Americans and costing about $635 billion, chronic pain continues to be a challenge of pandemic enormity for patients, for their families, for medical establishment, and for society as a whole (3). What causes otherwise healthy people to develop pain-related disability, to withdraw from their regular activities, and to cultivate attitudes filled with gloomy perspectives, complaining, misery, and suffering (4)?

An autobiographic book by Ruben Gallego entitled “White on Black” (5) describing the life of a patient afflicted with severe cerebral palsy may provide some insights into these conundrums. Notwithstanding the excruciating pain intensity (6) and the perception of blatant indifference from the nursing staff, the author only mentions “pain” twice throughout the entire text. In one instance, he routinely rolls out of bed to fall onto the floor in order to crawl to the bathroom. In the second instance, pain is likewise devoid of any fear or anxiety and is rather perceived as “senseless.” The author eventually graduates from a technical college, marries thrice and fathers three children. He figures out ways of refusal to accept fear and anxiety. Not so for chronic pain patients. They tend to catastrophize in conjunction with experiencing overwhelming anxiety about the ongoing and future pain episodes (7–9).

Chronic pain is indeed a complex phenomenon engaging, in addition to sensory systems, extensive threat response neurocircuitry with emotional and cognitive constituents merging on the brain networks comprising the nucleus accumbens (NAc), the amygdala, the extended amygdala, and the medial prefrontal cortex (mPFC). Assuming neurobiological overlap between the processing of pain and of other threatening signals the present article focuses on the components of pain that are related to fear and to anxiety and are germane for comorbidity of these conditions and for transition from acute to chronic pain (10).

At the outset of this review, we compare epidemiological and clinical data on pain and fear/anxiety comorbidity and this contrast serves as the foundation for the premise of a neurobiological similitude between these commonly comorbid conditions. We then discuss possible pain mechanisms as they relate to the mediation of fear and anxiety. Next, we depict specific evidence for testable hypotheses on mechanistically informed psycho-therapeutic and -pharmacological interventions. Finally, summary and conclusions are presented.

## Bibliographic Search

Preclinical and clinical English language peer reviewed literature search on anxiety and fear in pain along with the mechanisms of normative threat processing and their potential impairments in patients with chronic pain disorders was undertaken using PubMed (http://www.ncbi.nlm.nih.gov/pubmed) from inception until December 2017. Medical Subject Headings’ terms used included pain and nociception plus amygdala, anxiety, cognitive, fear, sensory, and unconscious. Information on the mechanisms and neurobiology of pain, fear, anxiety, cognition, analgesia, and salience were also drawn from recent seminal reviews of these topics (11–15). The scopes of the review were adjusted based on consultations with scientists and clinicians, manual searches for relevant articles from the selected papers’ reference lists along with the utilization of PubMed’s “similar articles” function.

## Key Terms

### Nociception and Pain

Noxious (mechanical, thermal, or chemical) stimuli activate C and A delta fibers. *Nociception* involves neurophysiologic mechanisms, including afferent activation in neural pathways responsible for detection or reflexive response to noxious stimuli. Like fear (see below), the response to an acute nociceptive stimulus includes pallor, freezing, tremulousness, diaphoresis, tachycardia, hypertension, and preponderance of the adrenomedullary, as compared to the noradrenergic stimulation (16, 17). The latter may be an adaptive reaction as epinephrine promotes memory consolidation (18) and improves coping with extreme situations by enhancing “gating” (i.e., activating descending modulatory systems) of noxious stimuli from reaching conscious awareness (1). Consequently, to assert prompt and automatic responses to hazardous situations, substantial nociceptive components remain sub- or unconscious (12).

*Pain* is experienced when the modulatory gating threshold is surpassed; so in acute situations, the attention is drawn to the bodily effects of the noxious stimuli. Chronic pain, by contrast, may occur in the absence of obvious tissue injury. Whatever the cause may be, pain is an unpleasant (1) or distressing (19) state associated with actual or potential tissue damage and comprised of sensory, emotional, cognitive, and social components (19, 20). Hence is the complex interplay among nociceptive perceptions and accompanying cognitive, behavioral, and emotional phenomena (13) so that pain experiences, derived from biological factors (e.g., genetic make-up, concentrations of endorphins and catecholamines, age, sex, underlying medical conditions and neuropsychopathology) are modulated by psychosocial variables (e.g., cultural, societal, and familial milieu in conjunction with upbringing; individual expectations; educational and professional backgrounds; and memories of prior pain episodes). In fact, the division between sensory and psychosocial pain expressions is not that perceptible (21) so co-occurring fear and anxiety not only contribute to the cognitive/behavioral pain aspects (22) but also worsen sensory phenomena (23).

### Fear and Anxiety

*Fear* is defined as a fundamental emotion promptly arising in the context of tangible and actual threats. It may be appropriate when reality-based and amenable to cognitive control, but is deemed to be a phobia (e.g., pain phobia or agliophobia) if becomes irrational (24). The emotional disturbance evoked by various threats is only part of the homeostatic regulation, with epinephrine secretion predominating that of norepinephrine in conjunction with the pallor, freezing, diaphoresis, tachycardia, hypertension, and shaking (17). *Anxiety* refers to a related yet distinct concept encompassing an uncertain source of threat (such as in chronic pain) or future-oriented cognitions linking fear and similar emotions to personal meaning of events and of actions (25, 26). Short-term anxiety states may be appropriate and adaptive whereas long-term ongoing anxiety periods are usually consistent with anxiety disorders (27). See Table 1 for comparison of acute and chronic pain with fear and anxiety.

**Table 1:** Key Characteristics of Pain, Fear and Anxiety.

	Threat			
	Clear		Uncertain	
Symptom (or intervening variable)	Fear	Acute pain	Anxiety	Chronic pain
Survival value	Avoidance of danger or injury		None	

Sensory input	Sensory overload: hearing, taste, sight, smell, and touch	Nociceptive overload	May be devoid of sensory or nociceptive input	

Unconscious component	Conditioned cues	Nociception	Libidinal drives	Irresistible drives to seek and consume analgesic drugs

Conscious component	Dread of loss of control and of dying	Distress and other negative affective states	Excessive worry and intrusive thoughts or memories	Unwarranted worry about an impending analgesic dose reduction and catastrophizing

Behavior	Avoidance, facial expressions, freezing, and escape	Escape from harmful agents or deterrence of motion to advance healing	Restlessness, fidgeting, and irritability	Pain behavior: facial expressions, stereotypic actions, complaining, and absenteeism; using pain as proxy for gaining pity, appreciation, or exemption from routing chores and responsibilities

Neuroanatomy	Lateral amygdala, CeA, NAc, BA	NAc, amygdala, cingulate, and insular cortices along with brain stem nuclei e.g., PAG	Amygdala and extended amygdala, including the BNST	

Neurochemistry	Sympathetic arousal: epinephrine>>norepinephrine		Allostatic load in the form of CRF, glutamate, norepinephrine and glucocorticoids	

Autonomic responses	Pallor, freezing, diaphoresis, tachycardia, hypertension, and shaking		↓ heart rate variability	

Typical conditions	Panic disorder	Mechanical, thermal, chemical, ischemic or inflammatory injury	Generalized anxiety disorder	Negative affective states: hyperkatifeia, neuropathic pain, excessive responses to painful (hyperalgesia) or even normally non-painful (allodynia) stimuli

*BA, basal nucleus; BNST, bed nucleus of the stria terminalis; CeA, central nucleus of the amygdala; CRF, corticotropin-releasing factor; NAc, nucleus accumbens; PAG, periaqueductal gray matter*.

### Conscious and Unconscious Processing

An attempt to integrate elements from psychological formulations of fear and anxiety symptoms into a neurobiological entity faces a major question: how to define “*unconscious*” as it relates to brain processes that do not produce conscious percepts. “Unconscious” is a somewhat clichéd entity given multiple definitions ranging Freudian Topographic Model, e.g., dreams, parapraxes, traumatic, and painful memories (28) to universal archetypal images by Jung (29), and the System One fast and intuitive thinking processes in Kahneman and Tversky’s Prospect Theory (30).

“Unconscious” may be defined from the cognitive, emotional, neurological, psychopathological, pharmacological, and legal perspectives (among other things). We address unconscious processing in conditions such as pain, anxiety and fear (31, 32) from the neurobiological standpoint. Specifically, we will consider high-amplitude low-frequency endogenous excitation of the limbic system normatively subordinated to the cortical default mode network containment as a valid version of “unconscious” (12, 15, 33–35). Although only one of many acceptable ways that “unconscious” might be conceptualized, this approach’s advantages include: (a) clearly defined neuroanatomical and electrophysiological criteria (33); (b) a firm foundation of cognitive neuroscience establishing links to the related memory and attention networks research foundation (36), and (c) its relationship to neuropsychopathology has been extensively accepted (33, 37).

## Pain and Fear/Anxiety: Epidemiological and Clinical Links

Numerous epidemiological surveys suggest that anxiety disorders are particularly prevalent in pain patients (11, 38, 39) and are associated with worsened functional outcomes (7). The most comprehensive of these studies, the US National Comorbidity Survey Part II, found the odds ratio of 4.27 for the association between chronic arthritic pain and anxiety disorders (40). Similar figures were reported for other patients with arthritis and for those with migraine, back pain (41), spinal pain (42), fibromyalgia (43), and the complex regional pain syndrome (44). Such findings are consistent with the international chronic pain and anxiety data from 17 countries (*n* = 85,088) in various parts of the world (45).

The mechanisms of the above links are likely to be bidirectional (46) and to involve environmental, psychosocial, and neurobiological causes (47). Four potential categories of interaction may (co)exist between pain and fear/anxiety (11), including: (1) causality; (2) mutual influence; (3) common predisposing factor; or (4) independence (i.e., no interaction). Accordingly, anxiety accompanied by depression (48) and by other negative affective and cognitive states (49) may be a direct *cause* for emotional pain, that is to say psychache (50) and/or to produce pain *via* excessive muscle contraction as well as *via* endocrine or other stress-induced pathophysiological end organ alterations (51, 52).

“*Mutual maintenance*” (i.e., influence) appears to be the most notable interaction. That is why, pain commonly (53, 54) arising in the context of abuse and violence (11) can become a conditioned stimulus eliciting fear and anxiety that in turn enhance subjective pain experience (55, 56) with concurrent avoidance of both pain- and fear-related situations and ensuing deterioration of both conditions (46, 50). In a course of pain and fear/anxiety sensitization, cross-sensitization might likewise occur. If that is the case, pain episodes could increase susceptibility to the development of fear and anxiety syndromes (or even trigger relapse) and *vice versa*. In view of that, “pre-pain” state may turn into a *bona fide* pain state as increased fear or anxiety serves as a tipping point disrupting the equilibrium. This is observed in acute exacerbations of pain by anxiety in healthy subjects (57) and by tempering anxiety with drugs (e.g., gabapentin) that have both anxiolytic (55, 58, 59) and analgesic (60) properties. Serotonergic impairments (61) may be a *common predisposing factor*. Another predisposing factor is a chronic or excessive use of opioid analgesics that amplify pain [*viz*., opioid-induced hyperalgesia and/or pain chronification (61)] on top of evocation of anxiety, fear, and of other negative affective states (62, 63).

Aside from the clinical impression, including the vignette in the Introduction Section (5), no studies to date have examined outcomes of pain conditions that are devoid (i.e., *independent*) from fear and anxiety. On the other hand, due to recurring stress accompanied by hopelessness, catastrophizing (57), horror (64), or avoidance of pain-related situations (11, 65), chronic pain may be viewed as a version of post-traumatic stress disorder (PTSD) (65, 66). Since the lack of peritraumatic fear and horror confers resilience to the development of post-traumatic symptomatology (DSM-IV-TR) (67, 68), it may also be plausible to find paucity of chronic pain cases in the absence fear and anxiety responses. Consistent with this assumption, timely and adequate peritraumatic analgesia may prevent the development of fear learning in both laboratory animals (69) and in humans (70). In short, understanding neurobiology of pain as it may relate to that of anxiety/fear could further support the former’s inclusion within the disorders of the threat response system (65, 71).

## Pain and Fear: Clinical Presentation and Homeostatic Role

Pain may indeed be considered within the spectrum of the fear/anxiety disorders, which is encoded in the “*persistently high level of anxiety*” criterion of the Diagnostic and Statistical Manual of Mental Disorders, 5th Edition (27) diagnostic category for Somatic Symptom Disorder with Predominant Pain in consort with “chest pain” and “muscle aches or soreness” respective criteria of panic- and generalized anxiety disorders. While no clinical studies specifically link the neural bases of pain and fear/anxiety, multiple lines of evidence suggest that pain is embedded within extensive threat response circuitry (72) that is critical for the survival of individuals and species *via* the evasion of real and/or perceived hazards. Thus, both pain and fear may be considered as intervening variables connecting threatening stimuli and consequent pathophysiological alterations with behaviors aimed at regaining the homeostatic equilibrium (73).

In chronic pain, tissue injury and other threats are not directly related to the above changes but are rather modulated by biopsychosocial variables as well as changes in brain plasticity that may alter an individual’s responsivity. Explicitly, while pain and fear/anxiety symptomatology are derived from precipitating factors, their clinical manifestations may be shaped by psychosocial setting, pre-existing neuropsychopathology and prior exposure to the same type of stimuli (see section Key Terms). Moreover, responses to various threats may be interrelated, be they sensory, visual, or interoceptive (72), which is critical for coordination, prioritizing, and selecting the most advantageous choices (74).

## Acute/Chronic Pain vs. Fear/Anxiety: Circuits and Functions

Responses to goal-objects comprise diverse informational features of the stimuli, events, or internal states. These features include but not limited to rate, incidence, proximity, timing, and quantity of the stimuli (44, 75). Evaluation of these informational features is critical for higher level cognitive and valuational processing with consequent behavioral choices. Multiple brain regions are involved in the assessment of informational features, their valuation, and probability estimates.

Sensory inputs concerning threats are relayed to the lateral amygdala and are subsequently conveyed from there to the central nucleus (CeA) and to the NAc after passing through the basal nucleus (BA) for generation of fear-related physiological (e.g., autonomic nervous system), emotional, and behavioral (e.g., freezing, escape, fight, and avoidance) responses. The CeA’s laterocapsular division (namely, the nociceptive amygdala) is involved in the descending pain modulation system (i.e., gating) determining pain-related affect, motivations, and behaviors by integrating nociception with interoceptive and environmental information with higher order cognitive percepts of objectives, their valuation, and context (76–78).

Fear dulls acute pain, which may be advantageous from a phylogenetic standpoint by promoting fight-or-flight operations; anxiety conversely worsens pain experience (79). In terms of neuroanatomy, fear vs. anxiety are differentiated by the engagement of the of the extended amygdala structure, the bed nucleus of the stria terminalis (BNST) (15) in the latter (but not in the former) perhaps by reason of inadequate extinction (80–82) and/or due to top-down suppression by the hippocampus (56) and by the mPFC (80, 83). In the bottom-up fashion fear- and anxiety-related subcortical limbic structures indirectly (15) affect lateral, medial, and insular cortices to contribute to respective conscious experiences (14).

**Figure 1:**
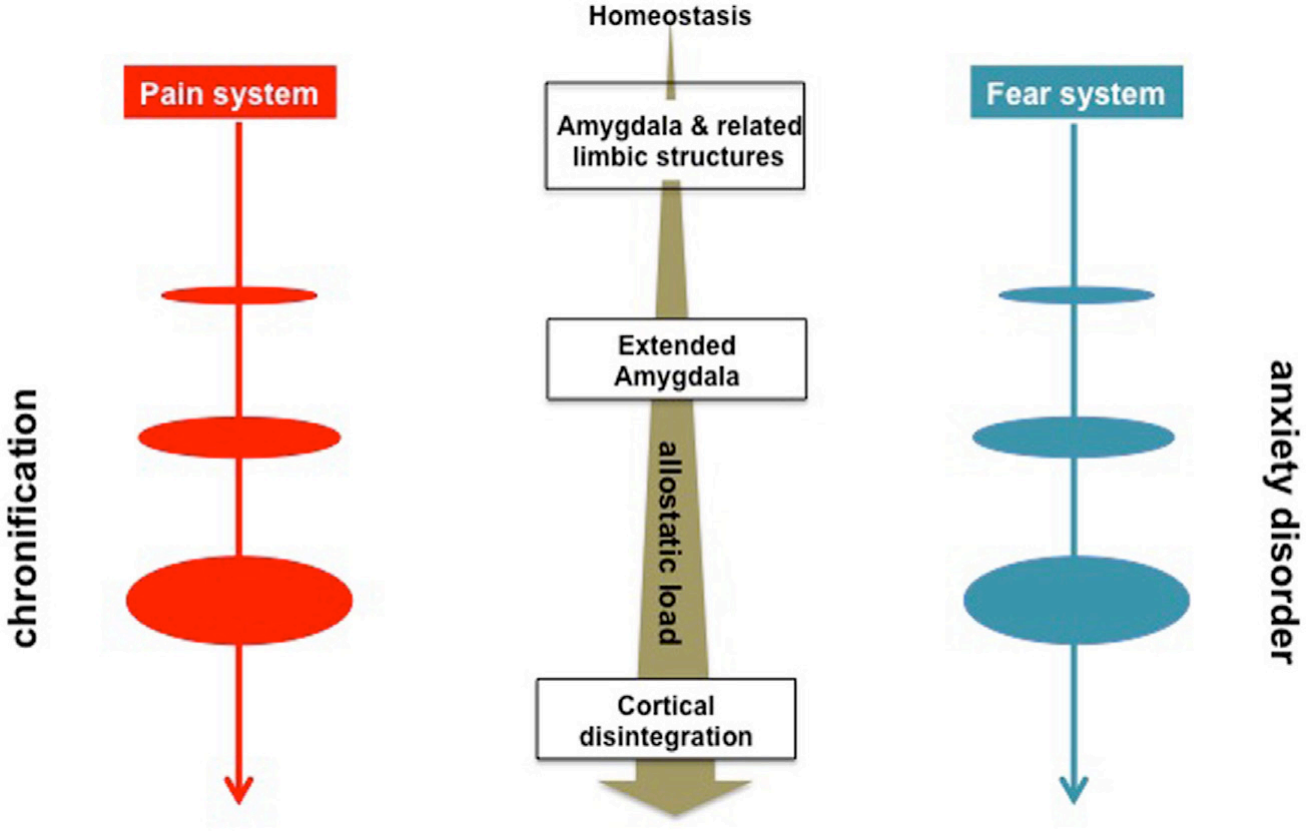
Schematic overview of the neurobiological processes underlying evolution of chronic pain and anxiety disorders. Sensory inputs concerning threats are relayed to the amygdala and other limbic structures, e.g., the central nucleus, the nucleus accumbens, the basal nucleus for generation of fear- or acute pain-related physiological (e.g., autonomic nervous system) behavioral (e.g., freezing, escape, fight, and avoidance), and cognitive responses (77, 78). Anxiety- and chronic pain symptomatology are characterized by the engagement of the of the extended amygdala structures, e.g., the bed nucleus of the stria terminalis along with allostatic load in the form of massive outpouring of stressogenic neurotransmitters and hormones manifested in negative affective states (10, 92).

Acute pain (84, 85) and fear (86, 87) are associated with phasic homeostatic responses to proximal and tangible threats. Chronic pain (88) and anxiety (15), in contrast, are of tonic and allostatic (89) nature with similar to each other autonomic changes (e.g., low heart rate variability) (90, 91); they are derived from uncertain threats and/or from contextual learning of the remote stimuli (Table 1). In psychological terms anxiety represents a failure to defend against unconscious libidinal drives (66). Acute pain with ensuing chronicity may be likewise conceptualized to be the limbic system’s respective failures to gate the nociceptive information from reaching conscious awareness and to properly process such information soon thereafter (12). Furthermore, recurrent dopaminergic trafficking consequent to ongoing pain episodes gives rise to between system “anti–reward adaptations” (88, 92–94) recruiting CeA, NAc, BA, and BNST that in concert contribute to allostatic load (Figure 1) in the form of massive outpouring of stress hormones *viz*., corticotropin-releasing factor, epinephrine, norepinephrine, glutamate, glucocorticoids, and pituitary adenylate cyclase activating polypeptide (95) manifested in fear, anxiety, and other negative affective states like those arising in the context of opioid overuse, that is to say, “hyperkatifeia” (63, 88, 92).

## Pain and Anxiety: Shared, but Separate Systems Contributing to Chronification

**Figure 2:**
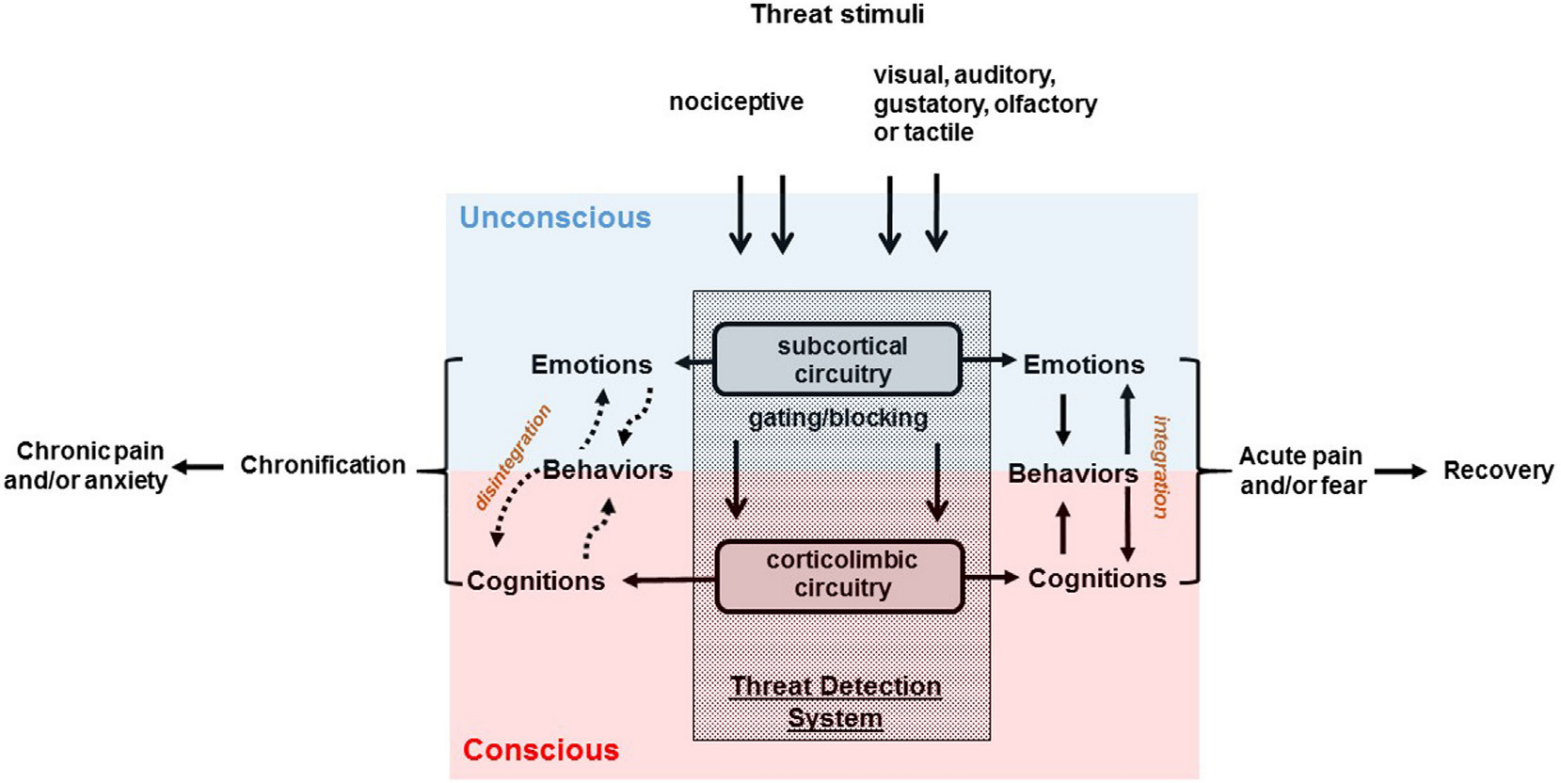
Schematic overview of the two-system construct underlying chronic pain and anxiety disorders. Sensory inputs of nociceptive nature may give rise to pain; while threatening visual, auditory, gustatory, olfactory, or tactile stimuli may bring out fear. Pain is conceptualized to be a failure of the limbic system to gate components of nociceptive information from reaching conscious awareness (12). In psychological terms, anxiety represents a failure to defend against unconscious libidinal threats (66). Chronic pain and anxiety symptoms may be attributed to a disintegration of two-system construct (15) comprised of subcortical circuits mediating unconscious threat-related physiologic, emotional, and behavioral responses in conjunction with linked, yet potentially independent higher corticolimbic network producing cognitive experiences.

Amygdala and related corticolimbic regions are conventionally considered to be the key component of the threat processing system involved in the experience of fear and it is commonly hypothesized that they simultaneously control conscious awareness of fear in conjunction with automatic defense responses (96). However, recent findings from human clinical (97, 98) and neuroimaging (99, 100) studies, as well as preclinical work (14), suggests that anxiety symptomatology may be attributed to a two-system construct (Figure 2) comprised of subcortical circuits mediating unconscious and automatic threat-related physiologic and behavioral responses in conjunction with closely linked, yet potentially independent higher corticolimbic networks producing conscious anxiety experiences with corresponding sets of drives and behaviors (14, 15). Their distinctiveness is supported by different temporal features of anxiety cognitions vs. automatic events (15, 101) c.f., preponderance of cognitive experiences lacking recognizable nociceptive input in chronic pain patients (102). The fact that conscious experiences of chronic pain (17) or of anxiety (103) are substantially more bothersome for patients than sensory percepts or defensive (re)actions potentially explains the relative inefficiency of analgesics that mostly target subcortical regions (104).

The respective contributions from the amygdala and from the extended amygdala to acute pain/fear and to chronic pain/anxiety are only modulatory and indirect (15) so that the circuits that are directly responsible for the subjective cognitions, valuations, and experiences may function independently from the subcortical limbic input. Such disintegration between cognition, perception, and emotions may occur due to (1) substantial dopaminergic surges in reward, motivation, and learning centers leading to “hardwired” neuroplasticity in the striato-thalamic-frontal cortical loop, with insuring top-down dissociation from the subcortical activity (105, 106); and/or (2) hypofunctionality of the excitatory glutamatergic afferents from the amygdala–hippocampus complex failing to produce bottom-up restrain of the striato-thalamic-frontal cortical loops (105, 107, 108).

Impartments of the bottom-up striato-thalamic-frontal cortical modulations may be observed in a number of neuropsychiatric conditions associated, such as pain (109), with heightened dopaminergic bursts in reward, motivation, and learning centers (88, 110, 111). For instance, positive symptoms of schizophrenia may become dissociated from the mesolimbic subcortical activity and persist notwithstanding presumably complete dopamine blockade by antipsychotic agents in about a quarter of psychotic patients (112, 113). Moreover, craving in patients addicted to opioids persists even in the face of fully occupied opioid receptors (114). This is also the case for cocaine craving in cocaine dependent subjects receiving agonist substitution therapy (115). Akin to drug addiction (116, 117), pain and anxiety chronicity lacking normal sensory input may be attributable to neuroplastic changes that become ingrained in the corticolimbic (e.g., thalamocortical, thalamo-striato-cortical, and amygdalo-cortical) synapses driving compulsive thoughts and repetitious actions (118).

## Diagnostic and Therapeutic Considerations

Psycho-diagnostic and -metric assessments may define and monitor cognitive neuroadaptational states, while neuroimaging combined with cognitive and biochemical challenges could be instrumental for demonstration of subcortical emotional and physiological aberrations. Thus, rather than targeting pain along the entire biopsychosocial continuum it may be useful to segregate this multidimensional system into cognitive, emotional, and sensory domains based on the distinct underlying circuitry. Addressing cognitive/subjective domain separately may provide a sound footing for understanding its role in the therapeutic armamentarium for chronic pain.

While none of the professionally delivered therapies for chronic pain appears to be superior, generic types of cognitive-behavioral techniques is the commonplace practice supported by clinical trials (118); other methodologies include motivational intervention, self-help and peer support. Suboptimal outcomes of these intervention call for a more personalized approach accounting for unique biological susceptibilities along with secondary gains (e.g., pain as proxy for gaining pity, appreciation, or exemption from routine chores and responsibilities), catastrophizing and other cognitive distortions and problematic decision-making processes.

Opioid analgesic agents improve sensory components of acute pain and their short term use in chronic pain (119) can ameliorate autonomic responses by aborting stress-induced catecholamines releases in part *via* blockade of the locus ceruleus activity (120, 121). These beneficial properties are, however, outweighed by severe side effects, including those resulting from opioidergic and dopaminergic stimulation with secondary worsening of reward and motivational deficits (109), as well as opioid induced endocrinopathies (e.g., hypogonadism) (122). Moreover, opioid analgesics providing instant pain relief (i.e., negative reinforcement) can become a conditioned stimulus eliciting future painful episodes. Opioid-induced changes in the mesolimbic dopaminergic pathway may underlie heightened incentive salience attributed to opioids or to related cues (i.e., drug craving) as well as the amplification of hyperkatifeia and of sensory pain components (63, 109). Overall, opioid analgesics possess advantageous therapeutic properties for the treatment of acute pain and for mitigation of traumatic memories. These qualities explain to some extent the rise of opioids as the drug of choice in the pharmacopeia of chronic pain. However, opioids’ efficacy has been questioned by modern (123) research suggesting relative inefficacy of these agents in up to 70% of patients in pooled analyses of rigorously designed clinical trials (124, 125).

There are other potential psychopharmacological strategies for the management of the reentrant (126) autonomous thalamocortical circuits (105). Anti-glutamatergic agents may be helpful (127) and have already been successfully used in pain patients (128–130). As an example, ketamine administration produces long-term analgesia lasting at least 3 months (131). The findings of neocortical/cortical glutamatergic desynchronization support this sort of strategies, but more research is needed for understanding cortical mechanisms of chronic pain (132). Glutamate inhibition may be also instrumental because of excitatory glutamatergic neurotransmission (133) sensitization arising in the context of stress-like anti-reward phenomena (92) resulting from pain-induced activation of dopaminergic pathways. Similar to chronic pain, PTSD’s cognitive symptoms of flashbacks and intrusive recollections may be temporarily disconnected from the anti-reward stress (27, 134) and respond to anti-glutamatergic agents (135). These findings add further support for the proposed and therapeutic strategies for chronic pain patients.

## Summary and Conclusion

Striato-thalamic-frontal cortical pathways coordinate motor, cognitive, and emotional functions within the brain, including regulation of fear- and anxiety-related amygdala activity (136) while the limbic system represents a set of subcortical and cortical structures engaged (among other tasks) in the processing of emotions, motivation, stress, and fear (137). This review compares the roles played by the systems above in acute/chronic pain and in fear/anxiety conditions to indicate that some features are shared. For example, there are parallels in the acute harm prevention motivation typical of both acute pain and fear. Conspicuous similarities between chronic pain and anxiety include lack of survival value, involvement of the adrenomedullary system, autonomic responses, the key role of the extended amygdala, and related limbic structures in the emotional/physiological components and disintegration of the cognitive and behavioral/physiological phenomena. On the other hand, nociceptive, neuropathic, immune, degenerative, traumatic, and malignant pain sources may be associated with diffused tissue damage (88), which is not typical of patients with fear or anxiety.

Although limbic system is commonly implicated in the pathophysiology of chronic pain syndromes (12), here chronic pain is also postulated to result from dissociation of limbic structures from physiologically linked striato-thalamic-frontal cortical pathways. Prefrontal cortex, amygdala, NAc, and thalamic nuclei are the key information hubs (138) and their firing/communication impairments underlie fundamental psychiatric symptoms involving perception, arousal, cognition, and emotions (139). Accordingly, the symptoms may be derived from top-down or bottom-up dysfunction or combination. Abnormal bottom-up activity results in excessive responses to painful (hyperalgesia) or even normally non-painful (allodynia) stimuli with corresponding deficiency in reward function and an overwhelming urge to eradicate pain (88, 92). These may become disintegrated from the top–down changes manifested in unrealistic and even catastrophic (140) expectation of continued pain and/or of unsuccessful analgesia, subjective pain overvaluation with regard to a subjectively acceptable amount of pain, i.e., framing (88).

Treatment with opioid analgesics does not adequately address such cognitive distortions and may even worsen them (109, 141). If corroborated by human studies, the abovementioned insights will have implications for the primary and secondary prevention of pain chronification. Identification of neurobiologic risk factors for chronic pain could lead to screening of vulnerable individuals. Those with heightened vulnerability owing to baseline anxiety symptomatology might be counseled to avoid prolonged pain exposure (primary prevention), or selected for early intervention (secondary prevention) even in the presence of mild warning signs (e.g., anxiety, drug seeking, and catastrophizing) early [<3 months (142)] in the course of the pain-related illness. The proposed model of chronic pain could also have treatment implications, as it supports the use of both psychosocial and pharmacological interventions for amelioration of chronic pain problems. Clinical experience suggests that utilization of such combined interventions is lower (143) than what could be projected from positive outcomes of clinical trials (144). And so, this review provides clinical researches and practitioners alike with the important knowledge base for understanding the rationale for anxiolytic therapy and raises their awareness of the unmet psychosocial needs of chronic pain patients. Lastly, this model may also provide important leads for recognition and treatment of pain problems in patients with other neuropsychiatric disorders, including schizophrenia, addictions, and major depression (109).

It is conceivable that future therapeutic interventions targeting pain will address somewhat independent emotional/sensory and cognitive/behavioral components. Patients will be then characterized according to this dichotomy and clear cut cases will be speared side effects by only receiving interventions aimed at the specific system. The time is probably ripe to commence clinical trials to pursue the presented ideas.

## Author Contributions

IE and DB conceived the idea, performed literature searches, and wrote the manuscript.

## Conflict of Interest Statement

The authors declare that the research was conducted in the absence of any commercial or financial relationships that could be construed as a potential conflict of interest.
